# Supplementary figures and images for: Regulation of gene expression under high hydrostatic pressure: the versatile role of the master regulator SurR in energy metabolism
Source: Front Microbiol. 2025 May 30;16:1593936. doi: 10.3389/fmicb.2025.1593936 (PMC12162507; doi:10.3389/fmicb.2025.1593936)

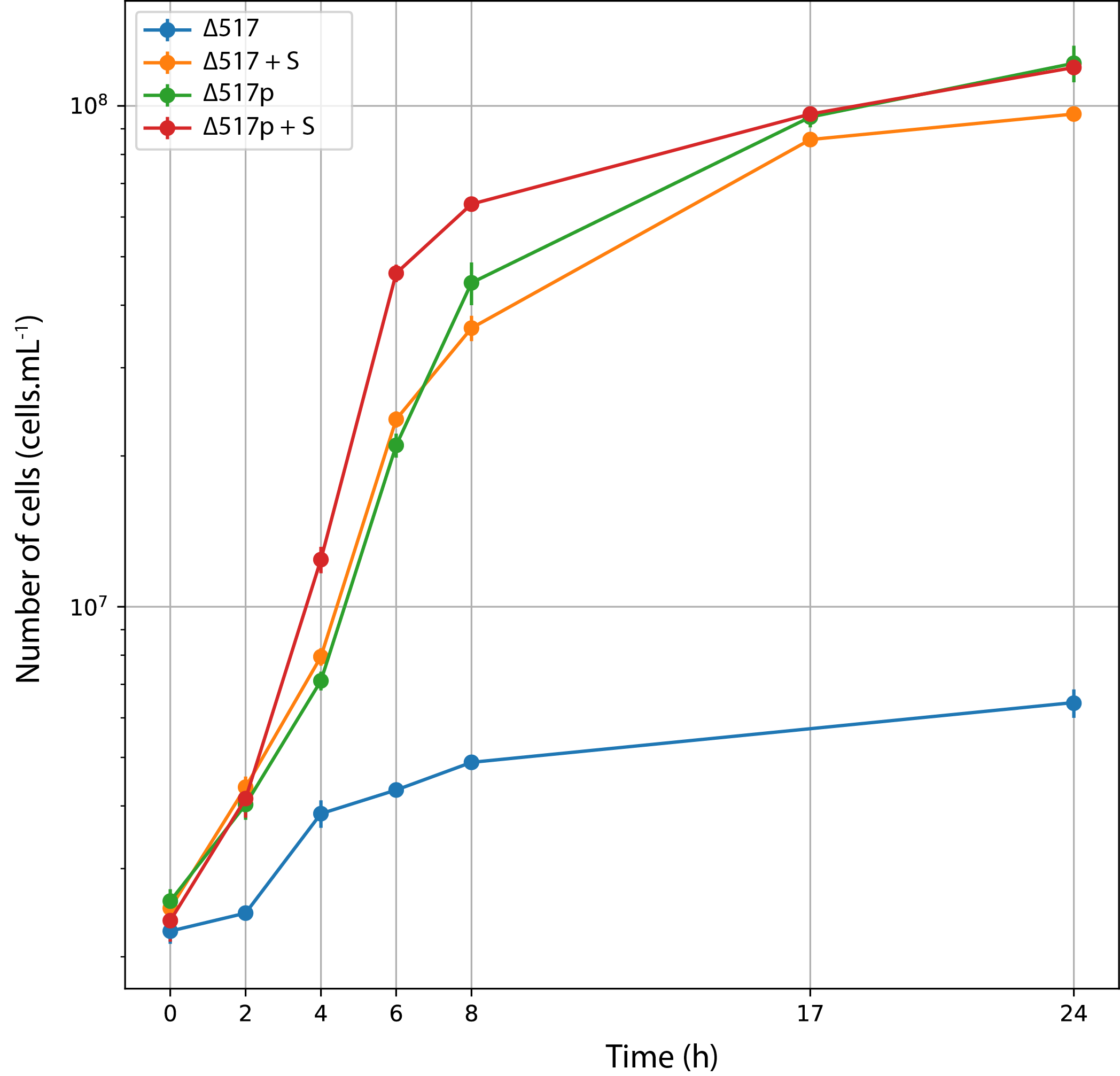

Supplement: SUPPLEMENTARY FIGURE 1 — Characterization of ∆517 strains with or without sulfur at 0.1 MPa. Growth assay were carried out at 85°C in TRM and TRMm media. TRMm containing Pyruvate (5g.L-1) was used for ∆517p strain growths. The ∆517p strain was previously adapted 96h in this medium before being subcultured for the growth kinetics. The growth rates are 0.059 h-1 (∆517, blue curve), 0.35 h-1 (∆517 + S, orange curve), 0.41 h-1 (∆517p, green curve) and 0.48 h-1 (∆517p + S, red curve). [file Image_1.tif]

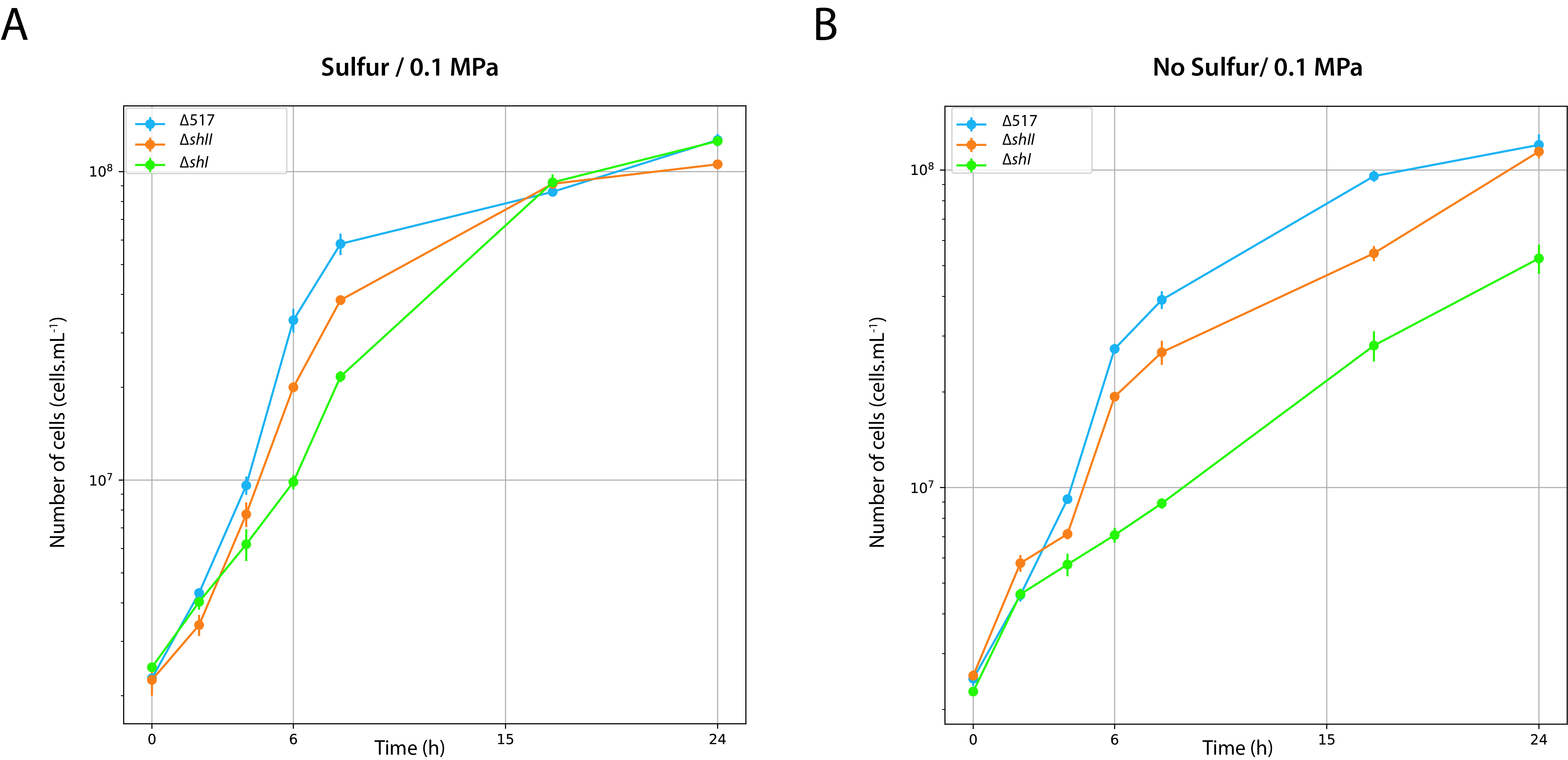

Supplement: Supplementary FIGURE 2 — Characterization of ∆shI and ∆shII mutants at 0.1 MPa. Growth assays were carried out in TRMm medium at 85°C, with sulfur (A) or without Sulfur (B). With sulfur, their respective growth rates are 0.41 h-1 and 0.28 h-1, while without sulfur, their respective growth rates are 0.33 h-1 and 0.11 h-1. [file Image_2.tif]
