# Supplementary material for: Regulation of gene expression under high hydrostatic pressure: the versatile role of the master regulator SurR in energy metabolism
Source: Front Microbiol. 2025 May 30;16:1593936. doi: 10.3389/fmicb.2025.1593936 (PMC12162507; doi:10.3389/fmicb.2025.1593936)
Supplement: Supplementary file 3 [file Table_1.docx]

TableS1: List of Strains used in this study

| Strains Names | UBOCC number | Genotype or other relevant characteristics | Genome region(s) deleted from parent strain | Source or reference |
| --- | --- | --- | --- | --- |
| ***E. coli*** |  |  |  |  |
| DH5α |  | *Φ80dlacZ*Δ*m15, recA1, endA1, gyrA96, thi-1, hsdR17 (r_k_−, m_k_+), supE44, relA1, deoR,* Δ*(lacZYA-argF)U169* |  | Thermo Fisher Scientific, Asnières, France  (Hanahan, 1983) |
|  |  |  |  |  |
| ***T. barophilus*** |  |  |  |  |
| ∆517 | UBOCC-M3300 | Δ*TERMP_00517* in TRM + Sulfur |  | Birien et al., 2018 |
| ∆517p | UBOCC-M3535 | Δ*TERMP_00517* in TRMm + Pyruvate |  | This study |
| ∆*mbh* | UBOCC-M3309 | Δ*TERMP_00517* Δ*MBH* in TRM + Sulfur | From TERMP_1471 to TERMP_1498 | This study |
| ∆*shII* | UBOCC-M3311 | Δ*TERMP_00517* Δ*SHI* in TRM + Sulfur | From TERMP_00067 to TERMP_00070 | This study |
| ∆*shI* | UBOCC-M3312 | Δ*TERMP_00517* Δ*SHI* in TRM + Sulfur | From TERMP_00536 to TERMP_00539 | This study |
| ∆*mbs* | UBOCC-M3313 | Δ*TERMP_00517* Δ*MBS* in TRM + Sulfur | From TERMP_00853 to TERMP_00865 | This study |
| ∆*surR* | UBOCC-M3533 | Δ*TERMP_00517* Δ*SurR* in TRM + Sulfur (incompleted deletion) | First 137bp of TERMP_00656 (nucleotides 563,829 – 563,965) | This study |
| **Plasmids** |  |  |  |  |
| pUPH |  | Pop-in Pop-out vector |  | Birien et al., 2018 |
| pUFH-2 |  | Cloning of homologous regions flanking *TERMP*_*00005* |  | Thiel et al., 2014 |
| pUFH-3 |  | pUFH-2 + *surR* |  | This study |
| p6MP-∆*mbh* |  | pUPH+ homologous regions flanking the  double clusters Mrp-Mbh1 and Mrp-Mbh2 |  | This study |
| p6MP-∆*mbs* |  | pUPH+ homologous regions flanking the   cluster Mrp-Mbs |  | This study |
| p6MP-∆*shI* |  | pUPH+ homologous regions flanking the cluster ShI |  | This study |
| p6MP-∆*shII* |  | pUPH+ homologous regions flanking the   cluster ShII |  | This study |
| p6MP-∆*surR* |  | pUPH+ homologous regions flancking the fisrt 137 bp of TERMP_00656 |  | This study |

Table S2: Primers used for genetic constructions

| Primers name | Sequences (5’->3’) | Utilization |
| --- | --- | --- |
| KpnI-DHyd-1up | AAAAAAGGTACCATAAGAATCTTGCCATAAGGGC | To delete the double cluster *Mrp-Mbh* from nt 1,278,922 to nt 1,295,504 |
| DHyd-1do | CAAATCAAGAGATGAGGTGAGAAAACTTTCCGCTTTAAGTTTCTTTTTATAATAA |  |
| DHYD-2-up | TTATTATAAAAAGAAACTTAAAGCGGAAAGTTTTCTCACCTCATCTCTTGATTTG |  |
| BglII-DHyd-2do | AAAAAAAGATCTTTTCTGCAAGCTCAATAGCTTC |  |
| Verif_Trans_Hyd_Up | TTTTCCTCCCCTGGAAACTTCTTCC | To analyze the deletion of *the double cluster Mrp-Mbh* |
| Verif_Trans_Hyd_Do | ATCGGAGGTGTAAGGAGAGATCTCAAG |  |
| KpnI-∆Mbs_1up | AAAAAAggtaccACGTCAACATTTATGAAGTAAAACGCTATCCT | To delete the cluster *Mrp-Mbs* from nt 736,353 to nt 746,804 |
| ∆Mbs_1do | CTTGGAGGGTTGGTATTATCTTTTTCTCTTTTCTTAGGCTTTTCTTACATGAGAAAAA |  |
| ∆Mbs_2up | AAGAGAAAAAGATAATACCAACCCTCCAAGAGTTTAAACTTGAAAG |  |
| BamHI_∆Mbs_2do | AAAAAAGGATCCGGGAAAGATTGAGCATTACTTTGATGAATATCCT |  |
| Verif_∆Mbs_YM_do | CTTCATATACCTGCACCTCAGCGT | To analyze the deletion of the cluster *Mrp-Mbs* |
| Verif_∆Mbs_YM_up | GCCGAGAGCTATTGGAATTCTGG |  |
| KpnI_∆SHII_1up | AAAAAAGGTACCGGCCTCTCTGGAGTTGGAACCCTCT | To delete the cluster *ShII*  from nt 59,761 to nt 63,613 |
| ∆SHII_1do | GAGAAATTTTGAATAGACGGGATCATCACCCGAAAGATTGCTTCTC |  |
| ∆SHII_2up | ATCTTTCGGGTGATGATCCCGTCTATTCAAAATTTCTCTATCTATTTTTGTTAAATC |  |
| BamHI_∆SHII_2do | AAAAAAGGATCCTCCATTTTGTTCTCGAGCATTAAAGCAACA |  |
| Verif_trans_SHII_up | TTCTGCAAGAGCTTAGGAACGCA | To analyze the deletion of the cluster *ShII* |
| Verif_trans_SHII_do | TATGCATCCGAGTTTCGATTTAACACATATAGC |  |
| KpnI_DSHI_1up | AAAAAAGGTACCCCCAATAAAATAAGCTCATTTATGGCAGCG | To delete the cluster *ShI* from nt 452,514 to nt 453,797 |
| DSHI_1do | GGGGAGGGGATCTCTATTCTCTTTTCTAAATTTTATCTCTGGTGGTTTTATGA |  |
| DSHI_2up | TTTAGAAAAGAGAATAGAGATCCCCTCCCCATGAACATCAT |  |
| BglII_DSHI_1do | AAAAAAAGATCTAAAGACATTTTCGAGCTCCTCATACCC |  |
| Verif_SHI_up | CCGGGCTCAAATTTTTCACTTAGAATCG | To analyze the deletion of the cluster *ShI* |
| Verif_SHI_do | CAATGAACATCATTTGTCCTTCGCCAC |  |
| KpnI_∆SurR_YM4-1up | AAAAAAGGTACCGTCTATAGCCGCTCGGGGAATATATTG | To delete partially the gene *SurR*  from nt 563,829 to nt 563,965 |
| ∆SurR_YM4-1do | TGAGTAGGTGGGGGACCCTCAAAATCATGGAAAGGGAGGGAC |  |
| ∆SurR_YM4-2up | CCCTTTCCATGATTTTGAGGGTCCCCCACCTACTCAAATTTGTAGC |  |
| BamHI_∆SurR_YM4-2do | AAAAAAGGATCCTAACATTTTTGCGAAAGCCATGGGAG |  |
| BamHI_SurR_int_up | AAAAAAGGATCCAACGGTACAAACTTGTTTAGCTCTTC | To analyze the partial deletion of the gene *SurR* |
| KpnI_SurR_int_do | AAAAAAGGTACCCATTCTGGGAAATAAGGTGAGGAGAGA |  |

Table S3: Primers used for the RT-QPCR

| **Targeted Gene/Cluster** | **Targeted Locus** | **Forward Primer (5’ to 3’)** | **Reverse Primer (5’ to 3’)** | **Expected Amplicon size (bp)** |
| --- | --- | --- | --- | --- |
| *sh*II | TERMP_00068 | TACCTACCGGCCAGGTCAGT | GGTCCCCGAA TGCCCACA | 180 |
| *sh*I | TERMP_00536 | GCATAGCTGACCC TTCCATTCTT | AACAACGCTGATCTGCTCTAT GG | 284 |
| *mbh*2 | TERMP_01494 | AGTGGCTTCTCCAAGGG TATCATAAC | ATCCTTGGTGTGCTCTTAATA GTAGCTAACC | 195 |
| *mbh*1 | TERMP_01480 | GTTCACGCTCTTCCTCCTTG | GTCGCTTCGCCAAGAGTATC | 174 |
| *mbs* | TERMP_00865 | GCAGCACTCTGTAG GCAACATC | TTCCTGACGAGCAGCCTTGAT C | 205 |
| *Sur*R | TERMP_00656 | CGTCCAGCTTTATGCCCCTGT   | CCTCAACGGCAGTCTCAAAAC A | 180 |
| *30S (S19)* | TERMP_00095 | AGTGGTGGCCGCTATTATTG | TAGGATTTCACCCCTACCCC | 156 |
| *30S (S13)* | TERMP_00128 | GGCATAAACTTTGCCACGATG GTG | TGCACCAATGAGGTGCATGTC | 195 |
| *pcna* | TERMP_00342 | GCATGAGGGCAATGGATCCA A | GGGTTACCTCAAGGAAGTTCT CCTCA | 198 |
